# Designing the syllabus for the EAP course “Architectural Art English”: A needs and genre analysis-based approach

**DOI:** 10.1371/journal.pone.0351750

**Published:** 2026-06-16

**Authors:** Rui Sun, Zhihao Chen

**Affiliations:** 1 Department of General Education, Hubei Institute of Fine Arts, Wuhan, Hubei, People’s Republic of China; 2 Wuhan Hikvision Technology Co., Ltd., Wuhan, Hubei, People’s Republic of China; Bahir Dar University, ETHIOPIA

## Abstract

This paper details the design and development of a discipline-specific English for Academic Purposes (EAP) syllabus for undergraduate students majoring in architecture and design related disciplines in an art school in China. Guided by the comprehensive needs analysis model established by Dudley-Evans and St. John [Developments in English for specific purposes: a multi-disciplinary approach. Cambridge University Press (1998)] and Swales [Genre analysis: English in academic and research settings. Cambridge University Press (1990)] genre theory, this study employed a university-wide questionnaire to gather data from 650 participants, including students and faculty. The needs analysis identified a critical disparity between students’ target academic aspirations—such as engaging in international collaboration, pursuing studies abroad, and understanding foreign lectures—and their present situational capabilities, particularly in listening comprehension and command of disciplinary vocabulary. In response to these findings, the proposed “Architectural Art English” syllabus is constructed around a genre-based, content and language integrated learning (CLIL) as well as task-based language teaching (TBLT) approach. It systematically incorporates seven key academic and professional genres relevant to the field: textbooks and monographs, research papers, case studies, and lecture scripts as input genres; and design statements, presentation scripts, and research abstracts as output genres. Each instructional unit is organized around a central architectural theme, utilizing authentic cases and genre analysis of move structures to make disciplinary discourse conventions explicit. The pedagogical framework emphasizes task-based learning, featuring authentic activities such as case analysis, design project presentations, and paper abstract writing to bridge the gap between language skills and disciplinary knowledge. This syllabus aims to addresses the complex interaction of learners’ target needs, subjective wants, and current proficiencies, facilitating their active participation in the global academic community of architecture and design.

## 1 Introduction

With increasing globalization of higher education, the demand for discipline-specific English proficiency has grown significantly. For college students, academic English is not an end itself but a means to an end. It is an essential medium for expressing concepts and engaging in professional cross-cultural dialogue. English for Academic Purposes (EAP) has thus emerged as a crucial bridge connecting professional knowledge with language skills.

English for Academic Purposes (EAP) refers to the teaching of English for academic study, including reading disciplinary texts, writing research papers, and participating in seminars [1]. It is distinguished from the broader field of English for Specific Purposes (ESP), which also covers vocational and professional contexts [2]. The present study is set within EAP scholarship as it focuses on preparing students for the academic communication demands of architectural art education. This framing informs the research questions guiding this study, which are presented in Section 4. In addition, the questionnaire design, drawing on established EAP needs analysis frameworks, was aligned with this academic focus.

The design of EAP courses requires careful attention to the specific literacy demands of learners’ target academic communities [1]. While EAP scholarship has extensively examined disciplines such as sciences, engineering, and economics, art and design disciplines remain significantly under-researched. This gap is particularly striking given the growing internationalization of art and design education.

Against this background, the present study investigates the design of an EAP syllabus for a university-wide elective for undergraduates at a Chinese art and design institute. Methodologically, it contributes by systematically integrating needs analysis and genre analysis, two frameworks typically employed in isolation, to develop an evidence-based course framework. The needs analysis identifies students’ target needs, difficulties, and preferences—informing unit topics, tasks, and skill weighting. The genre analysis reveals move structures of key architectural texts—shaping unit sequencing and literacy goals. Together, they ensure the syllabus is both responsive to learner needs and grounded in disciplinary discourse. This integrated approach offers a replicable framework for EAP practitioners.

## 2 Literature review

EAP courses set out to teach the language and communication skills that specific groups of language learner need or will need to function effectively in their disciplines of study [2]. The process of identifying these needs is termed “needs analysis” [2]. T. Johns and Dudley-Evans and many other researchers reached a general consensus that needs analysis is a defining feature of ESP and, within ESP, of EAP [3] and corner stone as it leads to a very focused course [4].

When planning a syllabus, a central issue to be considered is the what content should be included int the course [2]. And the data collected in the needs analysis process is the major source of information to be used in determining and refining the content.

The definitions of needs analysis have been hugely broadened with experience and research and become increasingly sophisticated. This part elaborates on the development in needs analysis and reviews some revolutionary figures and their models.

### 2.1 The emergence phase (1960s–early 1970s): register theory

In this period, English teachers, with very little knowledge of science and technology, had to teach science students English for their subject studies. Their perception of students’ needs was naturally influenced by the then well-acknowledged theories in linguistics and register analysis and thereby the focus of ESP course was mainly placed on language items of grammar and vocabulary [2].

Halliday’s revolutionary book *The Linguistic Sciences and Language Teaching* laid the foundation for register theory where he systematically examined how language varies according to situations [5]. His model demonstrated three key dimensions of “functional variety of language” that shape specialized language: field, tenor and mode. While it provided an empirical, linguistic basis for creating teachable syllabi focused on the specific language of target domains, it mistook a description of the language for a description of need because it focused only on lexical and syntactic features. Lukin et al. [6] argue that Halliday’s register framework fails to account for the dynamic, interactional aspects of language use.

### 2.2 Systematization and the shift in communicative skills (mid-to-late 1970s)

The revolutionary shift came in this stage with the birth of John Munby’s model, Communicative Needs Processor (CNP) [7], which was a monumental attempt to create a complete and systematic framework for specifying the uses of language that learners were likely to encounter in specific purpose situations [3] and it laid the foundation of Target Situation Analysis (TSA). Based on variables like participant, setting, purpose, and channel, it entails a detailed questionnaire describing a learner’s communication requirements whose output was a list of communicative events and the linguistic functions required to perform them. Groundbreakingly comprehensive as it was, it met strong criticism for firstly still placing heavy emphasis on linguistic forms and secondly only considering the target needs of learners and neglecting other requirements such as learners’ lacks and wants [3] and thirdly for being impractical and mechanistic because the model was so complex that it was almost impossible to implement fully.

### 2.3 Humanization and simplification: learner-centered refinements (1980s)

In reaction to the complexity of Munby’s model, this phase focused on pragmatic simplification and the learner’s perspective was incorporated in the models. Scholars such as Richterich and Chancerel [8] attached great importance to identifying both objective and subjective needs of learners, emphasizing their interests and expectations, alongside institutional requirements.

Hutchinson and Waters [9] proposed the influential *Learning-Centered Approach*, which made a clear distinction between Target Needs—further categorized into necessities, lacks, and wants—and Learning Needs, which concern factors like motivation, learning styles, and available resources.

The strength of their models is that they placed the learner at the center of the process. It made NA a more feasible and adaptable tool for classroom teachers as simpler and mixed-method approaches (questionnaires, interviews, observations were adopted.

### 2.4 Integration, multidimensionality, and technological expansion (1990s–present)

Since the 1990s, needs analysis has evolved into a more comprehensive and multidimensional process.

One of the most influential models was introduced by Dudley-Evans and St John [4]. This integrated framework combined Present Situation Analysis (PSA), Target Situation Analysis (TSA), Gap Analysis, Learning Needs, and the consideration of both objective and subjective needs. They also proposed triangulation concept, highlighting the significance of collecting data from multiple sources such as learners, teachers, and employers to ensure reliability. Furthermore, they added a dynamic component to the frameworks by Hutchinson and Waters, highlighting the ongoing nature of needs analysis and advocating continuous assessment. This concept was further developed by scholars like Long [10] and Hyland [1] to ensure the ESP courses were adaptable and effective by responding to learner feedback constantly and instantly. In addition, Michael Long promoted Task-Based Needs Analysis approach to identify the specific tasks a learner needs to perform [10], creating more relevance with syllabus design.

More recent scholarship has expanded needs analysis. Research on disciplinary literacies [11,12] has shifted attention from generic language skills to how students are socialized into the communicative practices of specific academic communities. This academic socialization perspective examines how learners acquire discipline-specific ways of reading, writing, and speaking through participation in their target communities. For architecture, this means understanding not only written genres but also spoken practices like design critiques and studio presentations which are often overlooked in traditional needs analysis.

Among the models reviewed, Dudley-Evans and St John’s [4] framework was selected for this study for several reasons. Unlike Halliday’s register analysis, which focuses narrowly on lexical and syntactic features, and Munby’s CNP, which is comprehensive but impractical and ignores learner perspectives, the Dudley-Evans and St John model integrates multiple analytical dimensions—target situation analysis, present situation analysis, gap analysis, learning needs, and professional communication information and can be easily carried out. It also incorporates triangulation and dynamic assessment, features absent in earlier models such as Hutchinson and Waters’ [9] learning-centered approach, which remains relatively static. For this study, which investigates the complex needs of art and design students across three stakeholder groups in a specialized institutional context, this model’s emphasis on multi-perspective data collection and its accommodation of genre analysis make it the most appropriate framework.

### 2.5 Genre analysis

Genre analysis provides a systematic framework for understanding the rhetorical structures of academic texts [13]. Swales’ move-step model has been widely applied to analyze research articles, theses, and other academic genres. Genres are the media through which members of professional or academic communities communicate with each other [14]. For architecture education, studies have examined genres such as design critiques [15] and studio presentations [16]. The pedagogical value of genre analysis lies in its capacity to make explicit the rhetorical conventions of target genres, enabling instructors to design tasks that scaffold students’ comprehension and production of these texts [17,18].

Contemporary EAP research has pushed genre analysis beyond text-based description, with the multimodal turn challenging traditional models of needs analysis [19–22]. This line of inquiry examines how meaning is constructed across visual, spatial, and verbal modes—a dimension particularly relevant for design disciplines where texts are rarely purely linguistic. Architecture students, for instance, work with drawings, models, digital renderings, and verbal presentations, requiring competencies that extend beyond traditional written texts. As Bradford [19] argues, EAP must address how students “combine multimodal literacy with other language-related competencies” to access disciplinary knowledge, largely absent from earlier frameworks. This multimodal perspective also connects to broader questions of academic socialization, as learners acquire not just textual patterns but the social practices and epistemological assumptions embedded in disciplinary genres [11,12]. Together, these developments highlight the need for EAP pedagogy to engage with the full complexity of disciplinary discourse.

## 3 Theoretical framework

### 3.1 Models by Dudley-Evans & St. John

One of the major purposes for this research is to run a pre-course needs analysis for the design of an EAP course syllabus for college students majoring in arts and design, primarily guided by the multi-faceted models established by Dudley-Evans & St. John [4]. These models are particularly appropriate because they are presented with more clarity and incorporate a wider range of crucial variables, essential for understanding the linguistic, communicative and subjective needs of learners in an EAP setting. More specifically, these models can assist the stakeholders of an EAP course in investigating the following key aspects:

Target situation analysis and objective needs: this element examines the tasks and activities learners are/will be using English for. In this case study, the research shall attempt to identify what tasks and what specific language and communication skills students will need to perform in architecture and design related settings.

Present situation analysis: this factor considers the current language proficiency of learners which will help the course designer to assess the gap between their present level of language competence and the required level in, in this case study, their future academic settings. This analysis is very instrumental for deciding on the degree of difficulty of the materials and tasks the learners will be engaged in.

Wants and subjective needs: these are factors affecting how learners learn such as their expectations for the course, reasons for taking the course and previous relative learning experiences. Understanding learners’ motivations and attitudes is crucial for creating a tailored EAP course which is both engaging and effective.

Learning needs: these examine the effective ways learners will learn to improve their language proficiency which are not included in many other models but is rather relevant to the framing of an EAP course. Proper teaching and learning methods can help build learner’ confidence and mastering language use more efficiently and effectively.

Professional communication information: this encompasses linguistic analysis, discourse analysis and genre analysis. This part adds to the richness of their model and will be an essential part of analysis for this research work. It tries to identify the different genres and typical linguistic features in a certain field through the analysis of classical written or listening materials. Its analysis helps triangulate the results gained through other means (questionnaires, interviews). For this research, genre analysis will constitute another key focus.

### 3.2 Genre analysis theory by John Swales

A genre can be described as the way people in a specific community typically get things done through written or spoken discourse [23]. A core principle of genre analysis assumes that communication within academic or professional disciplines is not a freeform activity, but is constrained by established, recognizable patterns of discourse. Such structurally codified texts constitute what is known as a “genre”.

Genre analysis aims to identify patterns underlying specific genres (text types), such as nursing care plans [2]. Its primary objective is to uncover the linguistic features, rhetorical structures, and organizational patterns that are repeatedly employed within a specific disciplinary community to achieve its particular communicative goals.

What are the specific steps to conduct a genre analysis? John Swales [13], widely regarded as the pioneer in the field, established the “Move-Step Model,” a framework that has become the most classic approach in genre analysis:

Genre: a recognizable communicative event—such as research article abstracts, the academic book review, and the job application letter.

Move：a “move” is a functional unit which contributes to the genre’s broader communicative goals. Each move is often in the form of a segment or paragraph and serves a distinct purpose. For example, in a research article introduction, “Establishing a Territory” constitutes a fundamental move.

Step：a “step” refers to the specific linguistic techniques used to fulfill the function of a move. Several steps combine to accomplish the objective of a single move. For example, to fulfill the move of “Establishing a Territory,” an author might use the steps of “outlining key theories” and “citing literature.”

While Swales’ theoretical framework has certain limitations such as its potential to suppress the creative and individual expression particularly valued in the arts and humanities, it has advantages in transforming course objectives from cultivating abstract “language competence” to shaping concrete “academic skills.” This shift is a revolutionary breakthrough and significantly enhances students’ learning motivation and the practicality of the course. Through systematic textual analysis, course design can be based on verifiable evidence rather than relying solely on the creator’s personal experience.

### 3.3 Conclusion

This study integrates needs analysis and genre analysis in syllabus design. The needs analysis, drawing on large-scale questionnaire data, identifies students’ target needs, difficulties, content preferences, and learning approaches which informs unit topics, task design, and skill weighting across the syllabus. The genre analysis examines architectural texts to reveal the genre types, move structures and rhetorical conventions of key disciplinary genres, informing and shaping unit sequencing and literacy goals. Together, needs analysis ensures the syllabus responds to what students actually need, while genre analysis ensures instructional content reflects authentic disciplinary discourse. This integrated approach offers a replicable methodological framework for EAP practitioners in disciplinary contexts.

## 4 Research questions

The principal research question for this study is how a needs-based EAP syllabus can be designed to effectively address the specific linguistic and communicative requirements of undergraduate students in a college of art and design.

The specific research questions below will help sort out the priorities and necessities as to what should be included in the course content and how they should be presented and taught to the learners. Those questions are designed under the guidance of the theoretical models by Dudley-Evans and St. John and the genre theory by John Swales as well as informed by other previous relevant researches since researchers can always build their own studies on and glean useful information or ideas from existing reports on similar EAP courses which have been set up before [2]. Those questions are as follows：(1) What knowledge or skills are required in architecture-related academic settings? (2) In which areas do students perceive they would encounter difficulties？(3) What knowledge or skills should students learn in this course? (4) What learning approach should this course adopt? (5) What genres are included in this course and what are their features?

By addressing these questions, this study aims to gain a comprehensive understanding of university students’ specific needs for this course. The findings will serve as an empirical foundation for designing a more targeted and effective syllabus

## 5 Research methods

### 5.1 Ethics Statement

This research project, “Designing the syllabus for the EAP course ‘Architectural Art English’: a needs and genre analysis-based approach,” was carried out in full compliance with ethical standards. The study design was formally reviewed and approved by the Academic Ethics Committee of Hubei Institute of Fine Arts.

The study used an online questionnaire, distributed through the Wenjuanxing platform. Participants were recruited between May 21 and May 28, 2025, and included university students and teachers aged 18–60.

All participants’ rights were respected and protected. Before starting the questionnaire, everyone read a clear explanation of the study’s purpose, how the data would be kept anonymous, and their right to stop at any time. Participants were informed that submitting the completed questionnaire would be taken as their agreement to have their anonymous answers used for this research. Because the survey was completely anonymous and low-risk, the ethics committee waived the requirement for written or verbal consent and approved this “submission as consent” approach.

We maintained strict confidentiality throughout the study. All data were analyzed and reported only as group information, ensuring no individual could be identified. This research was conducted with integrity, aiming to advance knowledge while fully protecting every participant’s rights.

### 5.2 Research respondents

We handed out questionnaires among three groups in the college where this EAP course is provided: undergraduate students, postgraduate students, and teachers. These three groups were intentionally included to enable triangulation of perspectives, a key principle of comprehensive needs analysis [4,10].

Undergraduate students represent the primary target population of the proposed EAP course. As the intended beneficiaries of the syllabus design, their perceptions of needs, difficulties, and learning preferences are essential for creating a learner-centered syllabus.

Postgraduate students were included because their more advanced experience with disciplinary academic discourse provides insight into how needs may evolve as students progress through their studies. Their perspectives help identify potential needs that undergraduates may not yet recognize but will encounter later in their academic studies.

Teachers were included to provide expert perspectives on the academic and professional demands of the discipline. Drawing on their experience with both disciplinary content and student learning patterns, teachers can identify needs that students may overlook, particularly those related to disciplinary expectations.

### 5.3 Questionnaire

This study primarily employed a questionnaire as its research method, inspired by the scholarly work of several researchers, including [24–26]. Before it’s used in a pilot testing, we sent it to three experts, one professor and one lecturer teaching the subject and one English professor, to help establish the content validity.

Then, a pilot version of the questionnaire was tested with 30 undergraduate students from the same institution taking this course. These pilot participants were excluded from the main study sample. They were asked to complete the questionnaire and provide feedback on item clarity, relevance, any ambiguous wording and time taken to finish it. Based on this feedback, we revised the wording of 12 items for clarity and reduced the questionnaire length from 46 to 34 items to reduce respondent fatigue.

The questionnaire was organized into five sections (see S1 File).

Section 1 contained a single item designed to identify the respondent’s role, categorizing participants as either teachers in architecture, design, or related fields, undergraduate students, or postgraduate students. This design facilitated comparative analysis, aiming to provide a deeper understanding of the distinct perspectives across these relevant groups. Section 2 comprised 12 items addressing the first research question, which explores student’ target needs. Section 3 included 6 items aimed at analyzing the challenges students face, essentially investigating their current proficiency levels and the gap between this and their target needs. Section 4 contained 11 items, focusing on pinpointing the specific course content—what students want to learn. Section 5 had 5 items, addressing the aspect of learning methods—how students want to learn to most effectively enhance their engagement and motivation.

Sections 2 and 4 could be further divided into sub-items covering various language skills and one other specific topics. In Section 2, items 2–1 and 2–2 pertained to reading, 2–3–2–5 to listening, 2–6–2–8 to speaking, 2–9–2–11 to writing, and the final item 2–12 referred to “studying abroad” as one of the contexts where learners will use English in the future. In Section 4, items 4–1 and 4–2 related to specialized architectural knowledge, 4–3 and 4–4 to reading, 4–5 to listening, 4–6–4–8 to speaking, and 4–9–4–11 to writing. This detailed breakdown of activities was included to enable students to make more intuitive, accurate, and comprehensive selections.

With the assistance of departmental leadership, colleagues, and student advisors, the questionnaire was distributed university-wide. A total of 650 valid responses were collected, comprising 41 from college teachers (CT), 485 from undergraduate students (US), and 124 from graduate students (GS). Distributing the questionnaire to all three groups was intended to gather perspectives on the course from different angles, thereby ensuring more objective and valid conclusions.

All items used a Likert 5-point scale. For Sections 2 and 4, the scale was: 1 = Very Unimportant, 2 = Unimportant, 3 = Neutral, 4 = Important, 5 = Very Important. For Sections 3 and 5, the scale was: 1 = Strongly Disagree, 2 = Disagree, 3 = Neutral, 4 = Agree, 5 = Strongly Agree.

### 5.4 Genre analysis

This methodology is central to EAP pedagogy because it enables instructors to demystify the “hidden curriculum” of academic and professional writing in a certain discipline for students. That is why it constitutes an essential approach to this research.

It was employed to address RQ5 (“What genres are included in this course and what are their features?”) and to inform syllabus design. The analysis was conducted in three stages.

First, a corpus of architectural texts was compiled from multiple sources, including textbooks and monographs, academic journals, professional websites, and lecture transcripts. Seven specific genres were identified: textbooks and monographs, academic papers, case studies, and lecture videos (input); design statements, research abstracts, and presentation scripts (output). For each of the seven genres identified through preliminary review, samples were collected.

Second, these texts were categorized according to their communicative purposes, resulting in two broad categories: input genres and output genres.

Third, following Swales’ [13] move-step model, representative samples of each genre were analyzed to identify their recurring rhetorical structures. The findings of these analyses, including detailed move structures for two core genres and pedagogical applications for all seven, are presented in Section 9.

## 6 Data analysis

The study employed SPSS for data analysis. For each item in the questionnaire, response proportions and means were calculated for the three participant groups (undergraduates, postgraduates, and teachers). Statistical test selection was guided by the research question type and data characteristics.

To examine whether the three groups differed in their perceptions, non-parametric tests were used because the Likert-scale data were not normally distributed, violating parametric test assumptions. The Kruskal-Wallis H test was applied to each item across Sections 2, 3, 4, and 5 of the questionnaire, followed by independent-samples t-tests for pairwise group comparisons.

To examine the relative priority of different dimensions, paired-samples t-tests were conducted. These tests were applied to Sections 2 and 4, where multiple coherent dimensions were present. In Section 2, five dimensions were compared: reading, listening, speaking, writing, and study abroad. In Section 4, five dimensions were compared: specialized knowledge, reading, listening, speaking, and writing. For Sections 3 and 5, which consisted of individual items rather than dimensional constructs, only descriptive statistics and between-group comparisons were applied. This two-tiered approach is standard practice in applied linguistics research [27].

To assess the reliability of the questionnaire, Cronbach’s alpha coefficients were calculated for each section using SPSS for the data in S1 Table. As shown in Table 1, the overall questionnaire demonstrated excellent reliability with a Cronbach’s alpha of 0.962 for all 34 items. Each section also showed high internal consistency: the English usage scenarios section (12 items) achieved an alpha of 0.942, the difficulties section (6 items) 0.903, the teaching content section (11 items) 0.937, and the teaching methods section (5 items) 0.854. All values substantially exceeded the acceptable threshold of 0.70.

**Table 1:** Reliability analysis of the EAP needs survey.

Question numbers	Research categories	Cronbach’s Alpha	Number of items
2.1-5.5	Syllabus for the course Architectural Art English	0.962	34
2.1-2.12	English usage scenarios	0.942	12
3.1-3.6	Difficulties students typically encounter	0.903	6
4.1-4.11	Teaching content	0.937	11
5.1-5.5	Teaching methods	0.854	5

## 7 Results and analysis

The results are organized by the four research questions. Between-group comparisons (Kruskal-Wallis and Mann-Whitney U tests) were conducted for all RQs to examine differences among undergraduates, postgraduates, and teachers. Paired-samples t-tests were additionally applied to RQ1 and RQ3, where multiple coherent dimensions required comparison of relative priorities (reading, listening, speaking, writing, and study abroad for RQ1; specialized knowledge, reading, listening, speaking, and writing for RQ3). For RQ2 and RQ4, which consisted of individual items rather than dimensional constructs, only between-group comparisons were conducted.

### 7.1 Knowledge and skill requirements in architecture-related academic contexts

As shown in Table 2, both students and teachers consistently recognize the importance of all 12 listed items, with mean scores all above 3.5 (ranging from 3.73 to 4.00). The highest-rated item is 2–8 “Communicating in English with international students, foreign instructors, or experts” with a mean score of 4, closely followed by 2–12 “Students’ need for studying abroad or academic visits” with a mean score of 3.94. This indicates that students and instructors perceive the strongest need for English skills in intercultural communication and overseas academic experiences. Consequently, the course design should attach huge importance to oral communicative activities.

**Table 2:** Knowledge and skill requirements in architecture-related academic contexts.

2. During the learning process in architecture, landscape architecture, and art-related majors, students often use English in various academic activities. Please evaluate the importance or frequency of the following English usage scenarios:	Respondents	1 (%)	2 (%)	3 (%)	4 (%)	5 (%)	Generally agree (%)	Average score (each group)	Average score(total)	Non-parametric test		US-GS		US-CT		GS-CT	
H-value	P-value	t	p	t	p	t	p
2−1 Reading English academic journals	US	2.9	3.1	23.1	35.1	24.2	59.2	3.98	3.89	9.080	0.011	2.721	0.007	2.250	0.029	0.573	0.568
	GS	8.9	9.7	12.1	45.2	22.0	67.1	3.66									
	CT	9.8	9.8	19.5	39.0	38.4	77.4	3.54									
2−2 Reading English research papers	US	2.7	2.7	20.6	35.7	24.2	59.9	4.04	3.92	18.929	0.000	3.360	0.001	3.295	0.002	1.405	0.162
	GS	8.1	11.3	12.1	44.4	24.4	68.7	3.65									
	CT	14.6	7.3	31.7	22.0	31.5	53.5	3.34									
2-3 Attending professional courses taught in English by Chinese instructors	US	4.3	5.2	27.0	32.0	19.4	51.3	3.81	3.73	11.521	0.003	2.814	0.005	2.203	0.033	0.698	0.486
	GS	4.0	14.5	27.4	34.7	17.1	51.8	3.51									
	CT	9.8	19.5	12.2	41.5	37.7	79.2	3.37									
2-4 Attending courses taught by foreign instructors	US	2.7	2.3	21.4	35.9	15.3	51.2	4.04	3.89	34.241	0.000	4.884	0.000	3.678	0.001	0.613	0.541
	GS	9.7	8.1	21.0	46.0	14.6	60.6	3.49									
	CT	7.3	14.6	26.8	36.6	35.1	71.6	3.37									
2-5 Listening to lectures by foreign experts in English	US	2.5	1.9	24.9	35.7	25.8	61.5	3.99	3.92	9.582	0.008	1.892	0.059	2.845	0.007	1.801	0.077
	GS	4.0	5.6	21.8	42.7	17.1	59.8	3.81									
	CT	14.6	4.9	22.0	41.5	34.0	75.5	3.41									
2-6 Orally analyzing famous cases in English	US	2.7	3.7	24.5	35.1	14.5	49.6	3.94	3.81	23.917	0.000	4.152	0.000	3.694	0.000	0.609	0.544
	GS	11.3	6.5	21.0	46.8	9.8	56.5	3.47									
	CT	9.8	9.8	26.8	43.9	35.5	79.4	3.34									
2-7 Orally presenting one’s own design work in English	US	3.1	3.1	20.2	38.1	14.5	52.7	4.00	3.86	32.581	0.000	5.104	0.000	2.856	0.006	−0.174	0.862
	GS	10.5	6.5	27.4	41.1	17.1	58.2	3.43									
	CT	7.3	14.6	19.5	41.5	40.0	81.5	3.46									
2-8 Communicating with international students, foreign instructors, or foreign experts in English	US	2.1	2.3	17.7	37.9	25.8	63.7	4.12	4.00	22.382	0.000	3.860	0.000	3.178	0.003	0.830	0.408
	GS	7.3	7.3	21.0	38.7	19.5	58.2	3.69									
	CT	7.3	14.6	17.1	41.5	31.8	73.2	3.51									
2-9 Writing design descriptions for works in English	US	3.1	5.2	24.3	35.7	22.6	58.3	3.88	3.82	6.023	0.049	1.860	0.063	1.919	0.061	0.866	0.388
	GS	6.5	5.6	23.4	41.9	26.8	68.8	3.69									
	CT	2.4	22.0	24.4	24.4	32.4	56.8	3.51									
2-10 Writing paper abstracts in English	US	3.3	3.7	27.0	33.6	23.4	57.0	3.88	3.84	2.919	0.232	0.728	0.467	1.892	0.065	1.471	0.147
	GS	3.2	8.1	16.9	48.4	29.3	77.7	3.81									
	CT	12.2	14.6	17.1	26.8	32.4	59.2	3.46									
2-11 Writing short essays or reports for course assignments in English	US	3.5	3.7	25.6	34.8	21.8	56.6	3.89	3.81	8.405	0.015	2.495	0.013	2.093	0.042	0.804	0.423
	GS	5.6	9.7	22.6	40.3	24.4	64.7	3.63									
	CT	9.8	12.2	24.4	29.3	37.9	67.2	3.46									
2-12 Students having needs for studying abroad or visiting scholar programs for further education	US	3.1	2.3	21.4	35.3	26.6	61.9	4.03	3.94	9.750	0.008	2.648	0.009	2.349	0.023	0.875	0.383
	GS	7.3	8.9	14.5	42.7	29.3	72.0	3.73									
	CT	9.8	12.2	22.0	26.8	29.3%	27.1	3.54									

Jointly ranked third (mean = 3.92) are 2–2 “Reading English research papers” and 2–5 “Listening to lectures by foreign experts in English,” highlighting the importance of reading and listening skills. Item 2–1 “Reading English academic journals” and 2–4 “Attending courses taught by foreign instructors” share the fourth rank (mean = 3.89), reinforcing these two receptive needs. The lowest rated item is 2–3 “Attending professional courses taught in English by Chinese instructors,” possibly because Chinese instructors can use Mandarin to clarify content in such contexts. The three groups all agree that writing is the least frequently used skill in these academic settings as the three items (2–9, 2–10 and 2–11) related to writing were ranked lower with mean scores of 3.84, 3.82 and 3.81.

Table 2 also reveals significant disagreement between students and instructors on nearly all items, with all p-values < 0.05 with only one exception of item 2–10 (p = 0.232). Pairwise comparisons show that the primary differences lie between undergraduates and postgraduates, and between undergraduates and teachers. In contrast, postgraduates and instructors demonstrate consistent alignment across all items (all p-values > 0.05). This likely reflects that postgraduates, being more deeply engaged in specialized academic contexts, have an understanding of academic requirements closer to that of teachers. T-test results further indicate that undergraduates assign significantly higher importance to all types of knowledge and skills than both postgraduates and instructors, suggesting that instructors and postgraduates may underestimate the actual English needs perceived by undergraduate students in their academic learning.

**Fig 1:**
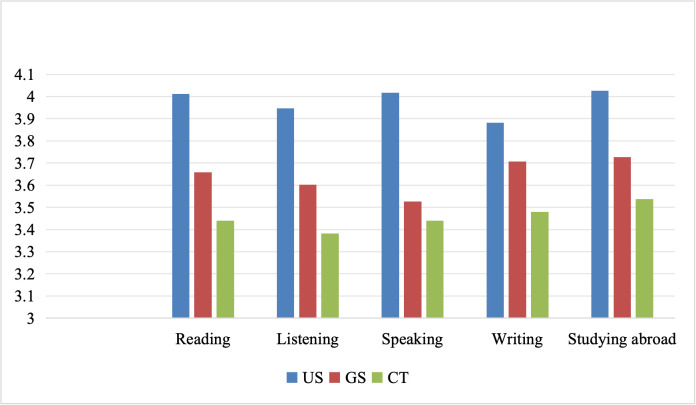
Group mean comparisons for skill importance.

The figure (see Fig 1) illustrates that undergraduates consistently rate all skills as more important than postgraduates and teachers, with the most pronounced differences observed in reading and speaking. Study abroad motivation shows a similar pattern, with undergraduates expressing the strongest interest in overseas academic experiences. (The corresponding items for each dimension can be found in Table 3).

**Table 3:** Comparison of knowledge and skill requirements in architecture-related academic contexts.

Pairs	Dimensions	t	p	5 Dimensions	Average score	Items
Pair 1	Reading	2.432	0.015	Reading	3.91	2−1 Reading English academic journals
	Listening					2−2 Reading English research papers
Pair 2	Speaking	2.672	0.008	Listening	3.85	2-3 Attending professional courses taught in English by Chinese instructors
	Writing					2-4 Attending courses taught by foreign instructors
Pair 3	Writing	−2.820	0.005			2-5 Listening to lectures by foreign experts in English
	Reading			Speaking	3.89	2-6 Orally analyzing famous cases in English
Pair 4	Listening	−2.782	0.006			2-7 Orally presenting one’s own design work in English
	Studying abroad					2-8 Communicating with international students, foreign instructors, or foreign experts in English
Pair 5	Studying abroad	1.494	0.136	Writing	3.82	2-9 Writing design descriptions for works in English
	Speaking					2-10 Writing paper abstracts in English
Pair 6	Speaking	−0.722	0.471			2-11 Writing short essays or reports for course assignments in English
	Reading			Studying abroad	3.94	2-12 Students having needs for studying abroad or visiting scholar programs for further education

Table 3 presents the results of the Paired-Samples T-Test conducted on the five dimensions of Section 2. The mean scores of these dimensions, ranked in descending order, are: studying abroad, reading, speaking, listening, and writing. This ranking aligns with the patterns discussed in the above analysis.

The data reveals that students exhibit strong motivation for overseas study ranking the highest at 3.94 and consistently perceive reading and speaking as significantly more important than writing. This perception is statistically confirmed by the p-values for speaking vs. writing (p = 0.008) and reading vs. writing (p = 0.005). Similarly, both studying abroad and reading are considered substantially more important than listening (p = 0.006 and p = 0.015, respectively). Conversely, no statistically significant differences were observed among studying abroad, speaking, and reading, indicating their comparable importance in respondents’ perception.

This preference pattern clearly demonstrates that students place greater emphasis on the communicative functions of English language learning, prioritizing interactive and academic mobility applications over more formal written output.

These findings have direct implications for syllabus design. The high priority given to oral communication and international academic experiences (items 2–8 and 2–12) suggests that the course should emphasize speaking tasks and include content assisting with cross-cultural communication. The consistently low ranking of writing indicates that while writing skills may not occupy a large percent but instead a smaller proportion of course time compared to speaking and listening.

### 7.2 Difficulties encountered in the learning process

As indicated by the combined proportion of “Strongly Agree” and “Agree” responses shown as “Generally agree” (>50%) (see Table 4), both students and teachers recognize that students face various difficulties and obstacles when learning architectural knowledge and skills. Furthermore, a comparison of the response proportions across the three groups reveals that students themselves have a significantly more pronounced perception of the difficulties they anticipate, as the student group’s agreement proportion is higher than that of the other two groups in nearly every item. This suggests that postgraduates and instructors relatively overestimate the current competences of undergraduate students.

**Table 4:** Difficulties encountered in the learning process.

3. In the learning process of architecture, landscape architecture, and art-related majors, what difficulties do students typically encounter? Please select according to the severity of the problem. (Teachers, please select based on your own observations; students, please select based on your own circumstances.)	Respondents	1 (%)	2 (%)	3 (%)	4 (%)	5 (%)	Generally agree (%)	Average score (each group)	Average score(total)	Non-parametric test		US-GS		US-CT	
H-value	P-value	t	p	t	p
3−1 Students find it difficult to read English textbooks, journals, and papers.	US	2.5	3.5	25.4	41.4	27.2	68.7	3.87	3.80	6.793	0.033	2.124	0.035	2.331	0.024
	GS	8.1	6.5	22.6	39.5	23.4	62.9	3.64							
	CT	12.2	12.2	22.0	31.7	22.0	53.7	3.39							
3−2 Students cannot keep up with the speech speed or understand the terminology when listening to lectures, courses, or videos by foreign experts.	US	2.3	3.9	20.8	40.6	32.4	73.0	3.97	3.89	8.472	0.014	2.099	0.037	2.665	0.011
	GS	8.1	8.1	16.1	38.7	29.0	67.7	3.73							
	CT	12.2	9.8	26.8	26.8	24.4	51.2	3.41							
3−3 Students have difficulty expressing themselves in English during class discussions, group presentations, design explanations, or when communicating with teachers and peers.	US	1.9	3.9	28.7	39.6	26.0	65.6	3.84	3.76	9.113	0.010	2.564	0.011	2.467	0.018
	GS	5.6	10.5	21.0	46.8	16.1	62.9	3.57							
	CT	7.3	19.5	19.5	36.6	17.1	53.7	3.37							
3-4 Students have difficulty writing English design descriptions, paper abstracts, English reports for class assignments, etc.	US	1.6	4.3	27.0	39.6	27.4	67.0	3.87	3.82	3.292	0.193	1.551	0.123	1.638	0.108
	GS	8.9	6.5	14.5	46.8	23.4	70.2	3.69							
	CT	0.0	17.1	34.1	22.0	26.8	48.8	3.59							
3-5 A major learning difficulty for students is the lack of professional English vocabulary and expressions related to architecture, landscape architecture, etc.	US	2.1	4.1	22.5	40.6	30.7	71.3	3.94	3.88	7.167	0.028	1.388	0.166	2.543	0.015
	GS	2.4	8.1	18.5	48.4	22.6	71.0	3.81							
	CT	4.9	22.0	12.2	43.9	17.1	61.0	3.46							
3-6 A major learning difficulty for students is the lack of professional knowledge in architecture, landscape architecture, etc., leading to insufficient content in their English expressions.	US	2.7	3.9	24.5	41.4	27.4	68.9	3.87	3.76	16.444	0.000	3.467	0.001	2.817	0.007
	GS	8.1	12.1	23.4	37.1	19.4	56.5	3.48							
	CT	7.3	14.6	24.4	41.5	12.2	53.7	3.37							

Among the items, 3−2 has the highest mean score (3.89), indicating that listening comprehension is the most challenging and weakest area for undergraduates. This finding presents an interesting paradox when considered alongside Section 2, where listening was ranked as the least important skill. Students recognize listening as their greatest weakness, yet they do not prioritize it in their perceived needs. This disconnect between “lacks” and “wants” has important pedagogical implications: the syllabus must not only handle listening difficulties but also help students recognize the importance of this skill.

The second-ranked difficulty is that students lack specialized English vocabulary and expression methods related to architecture (item 3–5). This highlights that even in an EAP course, which is fundamentally an English language course, a lack of professional vocabulary and unfamiliarity with disciplinary expressions can pose significant learning challenges. Therefore, it is necessary to incorporate content and activities focused on vocabulary and expression into the curriculum.

Data from the non-parametric tests in Table 4 show that Students and instructors consistently agree that students have difficulty writing English design descriptions, thesis abstracts, and course assignment reports. This aligns completely with common teaching experiences, as writing is also a major challenge for university students.

At the same time, for the vast majority of items (except for 3–4), the perspectives among the groups are inconsistent. Similarly, the differences primarily lie between undergraduates and postgraduates, and between undergraduates and teachers. The most significant discrepancy between undergraduates and both postgraduates and instructors occurs on the final item, 3–6 “A major learning difficulty for students is the lack of professional knowledge in architecture, landscape architecture, etc., leading to insufficient content in their English expressions”, with p-values of 0.001 and 0.007, respectively. The t-values, which indicate the magnitude of the difference, are 3.467 and 2.817—the highest values in their respective data columns. This indicates that undergraduates, on one hand, and postgraduates and teachers, on the other, hold completely different views regarding the importance of specialized architectural knowledge. Undergraduates are acutely aware that a lack of professional knowledge poses a significant obstacle to their English learning. In contrast, postgraduates and instructors, who already possess this knowledge, substantially underestimate its importance.

In summary, the analysis reveals a notable misalignment between perceived difficulties and prioritization of skills. The primary challenges—listening and writing—are precisely the skills assuming the lowest importance in the previous section. However, given that 73.6% of students consider attending courses taught by foreign instructors crucial for their academic development, listening should be repositioned as a secondary priority after reading and speaking. These findings directly shape syllabus priorities. The curriculum should accordingly incorporate modules to enhance listening comprehension capabilities. Furthermore, specialized knowledge in architecture must be integrated as a fundamental component of the course syllabus to address the identified gap in disciplinary content mastery. Several units, therefore, include structured lecture comprehension tasks with explicit attention to listening strategies. Lecture videos are also incorporated into the syllabus as significant teaching materials. The strong awareness of professional knowledge gaps among undergraduates (item 3–6) supports the integration of content knowledge throughout the course, with each unit in Part I introducing key architectural concepts and vocabulary before moving to language tasks, a finding that challenges the traditional separation between language instruction and content learning in EAP.

### 7.3 Perceptions on the importance of course content

Regarding the 11 teaching content items listed in Table 5, both students and college teachers consistently believed that all are important. Based on the ranking of mean scores for each item, the top two are: 4−2 “Mastering the core vocabulary and common terminology of architecture and landscape architecture” (3.90) and 4−1 “Learning the basic knowledge of architecture and landscape architecture” (3.88). This suggests that overall, students and instructors believe that mastering vocabulary, terminology, and professional knowledge related to architecture should occupy the most central position in the course syllabus, which is a complete departure from the structure of traditional college English courses. The lowest-ranked item is 4−11 “Writing English report assignments,” which aligns with the findings from Section 2, suggesting a general consensus on the relatively lower importance of writing. The third-ranked item is 4−4 “Understanding and analyzing the structure and content of English papers in the field of architecture and landscape architecture,” which relates to academic papers and shows that everyone recognizes the importance of research.

**Table 5:** Perceptions on the importance of course content.

4. What proportion do you think the following teaching content should occupy in the “Architectural and Artistic English” course? Please rate them according to their importance.	Respondents	1 (%)	2 (%)	3 (%)	4 (%)	5 (%)	Generally agree (%)	Average score (each group)	Average score(total)	Non-parametric test		US-GS		US-CT		GS-CT	
H-value	P-value	t	p	t	p	t	p
4−1 Learning the basic knowledge of architecture and landscape architecture.	US	1.6	2.7	22.5	45.2	28.0	73.2	3.95	3.88	8.449	0.015	2.962	0.004	1.283	0.206	−0.449	0.654
	GS	4.8	8.1	27.4	37.1	22.6	59.7	3.65									
	CT	0.0	19.5	14.6	39.0	26.8	65.9	3.73									
4−2 Mastering the core vocabulary and common terminology of architecture and landscape architecture.	US	2.1	2.3	21.4	45.2	29.1	74.2	3.97	3.90	6.483	0.039	2.149	0.033	2.297	0.027	1.129	0.261
	GS	3.2	12.1	19.4	37.9	27.4	65.3	3.74									
	CT	9.8	14.6	9.8	46.3	19.5	65.9	3.51									
4−3 Learning effective reading methods for English textbooks and professional journals.	US	1.9	3.3	23.1	43.3	28.5	71.8	3.93	3.84	10.769	0.005	2.713	0.007	2.775	0.008	1.008	0.315
	GS	9.7	8.1	19.4	37.1	25.8	62.9	3.61									
	CT	9.8	12.2	26.8	31.7	19.5	51.2	3.39									
4−4 Understanding and analyzing the structure and content of English papers in the field of architecture and landscape architecture.	US	2.1	2.9	22.5	44.5	28.0	72.6	3.94	3.87	8.212	0.016	1.461	0.146	2.810	0.007	2.015	0.049
	GS	3.2	6.5	25.0	38.7	26.6	65.3	3.79									
	CT	14.6	14.6	19.5	26.8	24.4	51.2	3.32									
4-5 Training listening comprehension of professional terminology by listening to lectures and watching videos.	US	2.1	3.3	24.3	44.3	26.0	70.3	3.89	3.81	6.305	0.043	3.127	0.002	1.007	0.320	−0.919	0.361
	GS	8.9	15.3	13.7	39.5	22.6	62.1	3.52									
	CT	2.4	17.1	14.6	39.0	26.8	65.9	3.71									
4-6 Analyzing international architectural or landscape design cases in English, and elaborating on design concepts.	US	1.6	2.7	24.1	44.3	27.2	71.5	3.93	3.82	15.221	0.000	3.679	0.000	2.499	0.016	0.137	0.891
	GS	6.5	13.7	23.4	34.7	21.8	56.5	3.52									
	CT	7.3	12.2	17.1	51.2	12.2	63.4	3.49									
4-7 Training for design project presentations and defenses in English, enhancing the logic and professionalism of expression.	US	2.5	2.1	24.5	43.3	27.6	70.9	3.92	3.85	3.641	0.162	2.388	0.018	1.024	0.311	−0.386	0.700
	GS	8.1	7.3	16.9	46.8	21.0	67.7	3.65									
	CT	4.9	9.8	19.5	39.0	26.8	65.9	3.73									
4-8 Organizing group discussions to practice design criticism and exchange of views in English.	US	1.6	4.1	27.2	41.6	25.4	67.0	3.85	3.78	3.343	0.188	2.087	0.038	1.782	0.082	0.734	0.464
	GS	8.1	6.5	21.8	42.7	21.0	63.7	3.62									
	CT	17.1	4.9	14.6	41.5	22.0	63.4	3.46									
4-9 Writing clear and structurally standardized English design descriptions.	US	2.3	2.5	23.5	43.3	28.5	71.8	3.93	3.84	8.703	0.013	3.038	0.003	1.928	0.060	0.284	0.777
	GS	7.3	9.7	20.2	41.9	21.0	62.9	3.60									
	CT	9.8	14.6	12.2	39.0	24.4	63.4	3.54									
4-10 Writing paper abstracts.	US	1.6	2.9	28.5	41.4	25.6	67.0	3.86	3.83	0.574	0.751	1.105	0.271	1.177	0.246	0.604	0.548
	GS	4.0	8.1	22.6	39.5	25.8	65.3	3.75									
	CT	12.2	9.8	14.6	31.7	31.7	63.4	3.61									
4-11 Writing English report assignments.	US	1.9	4.5	29.9	37.9	25.8	63.7	3.81	3.76	4.334	0.115	1.156	0.248	2.332	0.024	1.724	0.090
	GS	4.8	5.6	25.0	43.5	21.0	64.5	3.70									
	CT	17.1	12.2	17.1	31.7	22.0	53.7	3.29									

Divergent and Similar Views: Analysis of p-values from pairwise comparisons among the three groups reveals striking similarity on item 4–10 “Writing thesis abstracts” (p-values: 0.27, 0.246, 0.548). This indicates that regardless of academic stage, both students and instructors recognize the importance of writing thesis abstracts, further reinforcing the emphasis on research mentioned in the previous paragraph. As in previous sections, postgraduates and teachers held highly similar views on the vast majority of items, with all p-values exceeding 0.05. However, there were also disagreements among the three groups regarding the importance of teaching content, as non-parametric test p-values were <0.05 for 7 out of the 11 items (see Table 5). Disagreements between undergraduates and instructors occurred in 5 items which mainly focused on the dimensions of professional knowledge and reading, suggesting that instructors, due to their proficient mastery and application of knowledge, may overlook undergraduates’ deficiencies in these areas. The largest discrepancy in views between undergraduates and postgraduates was on item 4–6 “Analyzing international architectural or landscape design cases in English, and elaborating on design concepts,” where 71.5% of undergraduates considered this teaching content important, significantly higher than the proportion of postgraduates (56.5%).

**Fig 2:**
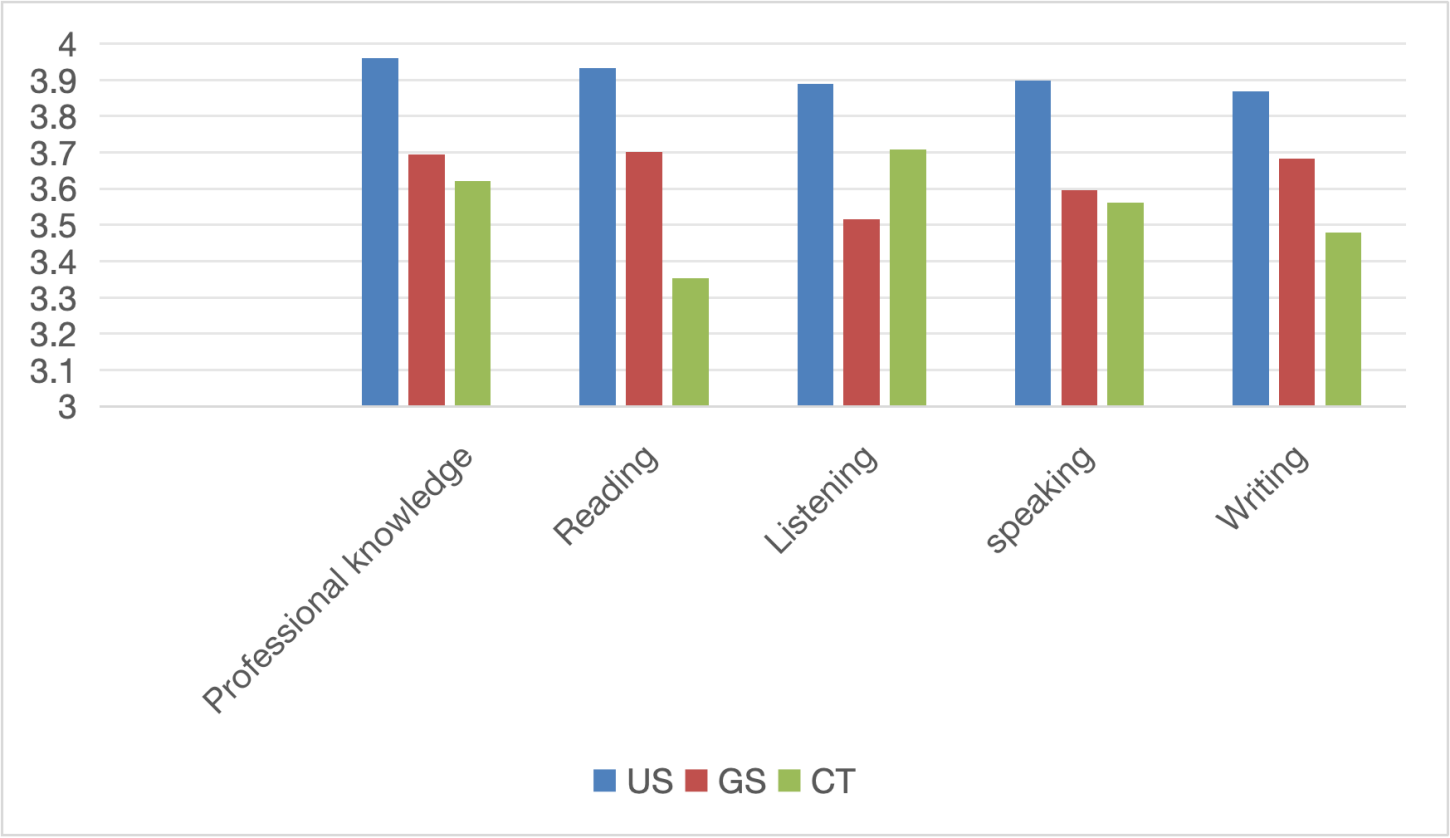
Group mean comparisons for course content preferences.

Section 4 also contains five dimensions: knowledge, reading, listening, speaking, and writing. The figure (see Fig 2) illustrates that undergraduates consistently rate all content areas as more important than postgraduates and teachers, with professional knowledge receiving the highest ratings across all groups. The most pronounced group differences are observed in reading and listening, where undergraduates’ mean scores substantially exceed those of teachers, suggesting an “expert blind spot” similar to that observed in Section 7.2. (The corresponding items for each dimension can be found in Table 6).

**Table 6:** Comparison of perceptions on the importance of course content.

Pairs	Dimensions	t	p	5 Dimensions	Average score	Items
Pair 1	Professional Knowledge	1.389	0.165	Professional Knowledge	3.889	4−1 Learning the basic knowledge of architecture and landscape architecture.
	Reading					4−2 Mastering the core vocabulary and common terminology of architecture and landscape architecture.
Pair 2	Reading	1.427	0.154	Reading	3.853	4−3 Learning effective reading methods for English textbooks and professional journals.
	Listening					4−4 Understanding and analyzing the structure and content of English papers in the field of architecture and landscape architecture.
Pair 3	Listening	−0.428	0.668	Listening	3.806	4-5 Training listening comprehension of professional terminology by listening to lectures and watching videos.
	Speaking			Speaking	3.819	4-6 Analyzing international architectural or landscape design cases in English, and elaborating on design concepts.
Pair 4	Speaking	0.457	0.648			4-7 Training for design project presentations and defenses in English, enhancing the logic and professionalism of expression.
	Writing					4-8 Organizing group discussions to practice design criticism and exchange of views in English.
Pair 5	Professional Knowledge	2.984	0.003	Writing	3.809	4-9 Writing clear and structurally standardized English design descriptions.
	Writing					4-10 Writing paper abstracts.
Pair 6	Reading	1.341	0.180			4-11 Writing English report assignments.
	Speaking					

The data (see Table 6) show that the mean scores for these dimensions, in descending order, are: knowledge, reading, speaking, listening, and writing. Except for the first dimension, the content and ranking of the remaining four dimensions are entirely consistent with Section 2, indicating that learning needs in academic contexts align closely with the importance of teaching content in instructional scenarios. Paired-sample tests on the five dimensions reveal a significant difference only between the knowledge dimension and the writing dimension (p = 0.003 < 0.05), indicating that a large proportion of respondents believed mastering knowledge is far more important than writing skills. For the other five paired comparisons, the t-values were all > 0.05, suggesting that the differences between their level of significance were not as substantial. To conclude, the syllabus should be designed to prioritize the four dimensions of knowledge, reading, speaking and listening over writing.

These findings directly inform content selection for the syllabus. The top ranking of professional knowledge and vocabulary (items 4−1 and 4−2) confirms that each unit should begin with knowledge-building tasks such as reading, vocabulary mapping and concept introduction. The high ranking of research-related content (item 4−4) supports the inclusion of academic paper analysis. The consistent emphasis on knowledge across all groups reinforces the decision to structure “Texts” for each unit around architectural history themes, ensuring that language learning is embedded in meaningful disciplinary content.

### 7.4 Views on teaching methods

Both students and teachers agreed that all five teaching methods listed are effective (see Table 7). One surprising finding was that the largest proportion of respondents across all three groups favored the same teaching method—5−3 “Case analysis”. The proportions of undergraduates, postgraduates, and teachers who selected “strongly agree” or “agree” for this method were 77.5%, 71.8%, and 63.4%, respectively. This indicates that the course syllabus should emphasize the use of cases and related exercises.

**Table 7:** Views on teaching methods.

5. Which of the following teaching methods do you think should be adopted in the course? Please select according to their effectiveness. (Teachers, please select based on your own observations; students, please select based on your own preferences.)	Respondents	1 (%)	2 (%)	3 (%)	4 (%)	5 (%)	Generally agree (%)	Average score (each group)	Average score(total)	Non-parametric test		US-GS		US-CT		GS-CT	
H-value	P-value	t	p	t	p	t	p
5−1 Teacher-led offline lectures.	US	0.8	2.7	19.4	43.5	33.6	77.1	4.06	3.98	9.528	0.009	3.013	0.003	2.228	0.031	0.687	0.493
	GS	4.0	11.3	15.3	44.4	25.0	69.4	3.75									
	CT	9.8	9.8	19.5	31.7	29.3	61.0	3.61									
5−2 Blended online and offline teaching.	US	2.5	2.7	21.0	42.3	31.5	73.8	3.98	3.87	20.262	0.000	3.842	0.000	2.754	0.008	0.552	0.582
	GS	5.6	9.7	25.0	41.1	18.5	59.7	3.57									
	CT	12.2	2.4	26.8	43.9	14.6	58.5	3.46									
5−3 Case analysis: analyzing classic or international architectural project cases to understand their design concepts and expression methods, thereby enhancing the practical application ability of professional English in architecture.	US	2.1	1.4	19.0	47.0	30.5	77.5	4.02	3.96	6.655	0.036	2.410	0.017	1.860	0.070	0.519	0.604
	GS	5.6	4.8	17.7	49.2	22.6	71.8	3.78									
	CT	4.9	12.2	19.5	36.6	26.8	63.4	3.68									
5−4 Design presentation simulation: simulating design presentation scenarios in class or competitions, training students’ ability to introduce design concepts, methods, and highlights in English.	US	1.9	3.1	20.4	45.4	29.3	74.6	3.97	3.89	10.430	0.005	2.652	0.009	2.381	0.022	0.907	0.366
	GS	4.0	12.1	16.9	44.4	22.6	66.9	3.69									
	CT	4.9	14.6	34.1	17.1	29.3	46.3	3.51									
5−5 Group collaborative presentations: organizing students to conduct English resource research, project presentations, and outcome reporting in groups, honing teamwork and English expression skills.	US	2.5	4.9	21.6	43.3	27.6	70.9	3.89	3.78	13.908	0.001	3.343	0.001	2.442	0.019	0.701	0.484
	GS	6.5	13.7	20.2	41.1	18.5	59.7	3.52									
	CT	12.2	14.6	22.0	26.8	24.4	51.2	3.37									

Another equally surprising finding was that across all three groups, the highest proportions of respondents who selected “strongly disagree” were for the last teaching method—5−5 “Group collaborative presentations: organizing students to conduct English resource research, project presentations, and outcome reporting in groups, honing teamwork and English expression skills”. The proportions were 7.4%, 20.2%, and 26.8%, respectively. This finding is also consistent with the p-values from the non-parametric test.

The p-values in Table 7 suggest that the most favored teaching method is 5−1 “Offline lectures” (mean score: 3.98), closely followed by 5−3 “Case analysis” (3.96). These are followed by two methods with close scores: 5−4 “Design presentation simulation” (3.89) and 5−2 “Blended online and offline teaching” (3.87). This highlights a general preference for traditional teaching methods, with relatively lower acceptance for newer approaches such as group presentations. The reasons for this may be the significant pressure and considerable time consumption associated with such methods, as well as perceptions among teachers of the low efficiency of them.

Based on the paired-sample test results across the three groups, undergraduates showed significant differences in perspective compared to postgraduates and teachers. The most notable difference was observed regarding 5−2 “Blended online and offline teaching” (p-values < .001 and 0.007, respectively). Postgraduates and teachers appeared noticeably more conservative in their attitudes. This may be related to the limited prevalence of such teaching methods, as only a small number of college courses have adopted this method, resulting in lower familiarity. In contrast, undergraduates may have more exposure to innovative teaching approaches, making them more familiar with or curious about such methods.

These findings directly shape the pedagogical approach of the syllabus. Preferences in teaching methods reflect partly the learning needs, which should be correspondingly integrated into the design of the curriculum. Based on the data analysis presented, instruction can adopt a blended online and offline model, while the content should incorporate case studies, design presentations, and a limited number of group collaborative activities.

## 8 Discussion and analysis

### 8.1 Responding to research questions

This section interprets the key findings and echoes the Research questions of this paper through the lens of Dudley-Evans & St. John’s [4] multidimensional needs analysis model, which distinguishes between target needs and learning needs, as well as between necessities, lacks, and wants.

RQ1: What knowledge or skills are required in architecture-related academic settings?.

The findings reveal that students’ target needs (wants) exhibit strong international and communicative characteristics. The highest-rated items, “communicating with foreign experts in English” and “pursuing further studies abroad”—indicate that students perceive oral communication and cross-cultural academic mobility as the most critical skills for their future.

Interestingly, while “attending lectures by foreign instructors” was considered crucial, the skill of “listening” itself was ranked relatively low in importance, despite being identified as students’ greatest difficulty. This contradiction reveals a mismatch between wants (what students think they need) and lacks (what they actually struggle with). The syllabus must therefore address not only students’ articulated wants but also their unacknowledged lacks.

RQ2: In which areas do students perceive they would encounter difficulties?.

Students identified listening comprehension as their most significant challenge (item 3–2), followed by lack of specialized vocabulary (item 3–5) and insufficient professional knowledge (item 3–6), reflecting students’ lacks: the gap between their current proficiency and the demands of target academic situations.

The data also revealed that undergraduates perceive these difficulties more acutely than postgraduates and teachers, suggesting an “expert blind spot” phenomenon—those who have already mastered disciplinary knowledge may underestimate how challenging the learning process feels to undergraduates. This finding highlights the importance of conducting present situation analysis alongside target situation analysis, as the two perspectives (learners vs. experts) can differ substantially.

RQ3: What knowledge or skills should students learn in this course?.

The data from Section 4 of the questionnaire indicate that students prioritize professional knowledge and vocabulary above all other content areas. Items 4−1 (“Learning the basic knowledge of architecture”) and 4−2 (“Mastering core vocabulary and terminology”) received the highest ratings, suggesting that students view EAP not as general language advancement but as preparation for disciplinary engagement.

This finding challenges the traditional separation between language instruction and content learning in EAP. As Bhatia [28] argues, professional discourse competence involves not only language skills but also an understanding of disciplinary culture. From a genre theory perspective, mastering a discipline means gaining access to its discourse community—learning not just linguistic forms but the rhetorical conventions, and social practices of that community. The syllabus therefore adopts a content-language integrated approach, with each unit introducing key architectural concepts and genres before moving to language tasks.

RQ4: What learning approach should this course adopt?.

Students expressed clear preferences for teaching methods. “Case analysis” emerged as the most widely accepted method across all groups, reflecting its alignment with the cognitive characteristics of architecture and design disciplines, which emphasize learning through concrete, authentic visual cases. This preference supports the use of case-based tasks throughout the syllabus.

Furthermore, students demonstrated a higher acceptance of “blended online and offline instruction” compared to teachers. Curriculum design should actively explore the integration of online resources such as recorded lectures to meet students’ expectations for flexibility and richness. This also aligns well with the popular Task-based Language Teaching advocated widely now in the English educational arena as offline teaching is designed to center around tasks students are expected to fulfill.

### 8.2 Implications for syllabus design

Based on the findings of this study, it is evident that the Architectural Art English syllabus requires deep integration across three dimensions: specialized content, linguistic knowledge and professional communication.

Specifically, the syllabus should adopt a content and language integrated approach, making architectural knowledge and vocabulary the core vehicle for language instruction. Through task-based teaching methods, it should create authentic professional scenarios such as architectural design presentations and project discussions for language practice. Case analysis should serve as the primary teaching method to naturally bridge the learning of architectural expertise with the development of professional communication skills.

Therefore, it is recommended to structure the curriculum as an organic framework organized around architectural themes as units, professional cases as the main thread, and practical tasks like design presentations and project discussions as the driving force.

## 9 Genre analysis

Through the analysis of the questionnaire survey, a preliminary framework for the “Architectural Art English” course has been established. The next step involves conducting textual analysis following the procedures outlined in genre analysis theory to develop the specific content of this framework, ultimately forming the syllabus for this EAP course.

### 9.1 Constructing the core genre framework

For EAP courses, identifying and systematically analyzing the core genres of the target discipline is essential to ensure the alignment of teaching content with learners’ authentic needs [2]. Based on the needs analysis model by Dudley-Evans & St. John [4], this study has identified the multidimensional needs of students enrolled in an art school for English skills in academic contexts. To translate these needs into teachable and learnable specific content, this chapter will construct a core genre framework for the course. This framework aims to systematically cover typical linguistic patterns and communication tasks in the field of architecture, providing a clear content blueprint for subsequent textbook development and classroom teaching.

The course syllabus derived from the previous chapter constitutes an organic framework structured around professional themes as units, case analysis as the main thread, and communicative tasks (such as presentations and discussions) as the output-driven component. The research also made efforts in collecting as comprehensively as possible the existing materials from multiple sources such as books, magazines, websites, literature and reached a conclusion about the major relevant genre types. Based on those, the present study categorizes the core English genres in architecture into two domains: input and comprehension genres, and output and expression genres. This classification reflects the natural sequence of language skill acquisition from reception to production.

#### 9.1.1 Input and comprehension genres.

This category forms the basis for learners to build their disciplinary knowledge system and internalize professional discourse patterns. This course focuses on the following four core genres:

1. Textbooks and monographs: as systematic carriers of disciplinary knowledge, their language is characterized by clear definitions, rich explanations, and strong logical coherence. The curriculum will derive the professional knowledge module from this, helping students build vocabulary networks and master professional expressive patterns.
2. Academic papers: particularly the abstract sections serves as windows for students to access cutting-edge research and academic research discourse paradigms.
3. Case analyses: such genres are widely found in textbooks and academic journals, aiming to illustrate design theories, strategies, or historical contexts through specific projects. The teaching focus is on cultivating students’ ability to deconstruct analytical texts and learn how authors integrate project descriptions, theoretical applications, and critical evaluations.
4. Lecture videos by International Scholars: these resources often include open courses or academic lectures on certain forums, featuring more colloquial language, frequent use of connectors, and examples to explain complex concepts. The teaching focus is on training students’ listening and information extraction skills, helping them master the structure of academic oral presentations and project reports and enhance their cross-cultural communication skills.

#### 9.1.2 Output and expression genres.

Developing learners’ proficiency in utilizing these genres is the ultimate objective of this course. The course will focus on training the following three output genres:

1. Design statements: this is the most representative written communication genre in architectural practice whose communicative purpose is to clearly articulate design concepts in order to convince the audience to of the value of the design proposal. Most contemporary architecture schools publish outstanding student design statements which can be designed as classroom translation tasks. Leading architecture firm websites such as Foster + Partners provide professionally written project descriptions in concise English, making them ideal reading and translation materials. Architectural publications including Architectural Review, Dezeen, and ArchDaily contain a rich collection of well-written English project descriptions.
2. Research abstract: academic journals like Journal of Architecture and Journal of Architectural Education provide downloadable English abstracts suitable for teaching materials. Lectures will concentrate on the instruction on the moves and steps in summarizing a study as well as the typical linguistic features of research abstracts.
3. Presentation scripts: Videos featuring “architecture student presentation” can be found on YouTube from international architectural lectures which can be perfect source of presentation scripts. This trains learners to integrate visual carriers with oral narration through the analysis of the persuasive narrative logic.

In conclusion, this core genre framework provides clear anchors for the course content. Course units will be progressively arranged according to an input-to-output sequence which ensures the synchronous accumulation and spiral progression of language skills and professional knowledge. In the following part, an example analysis on the first genre, textbooks and monographs, will be demonstrated to make clear the process and the result will be integrated into the final product, the syllabus of the course.

### 9.2 Analysis of textbooks and monographs

#### 9.2.1 Themes of textbooks and monographs.

By examining current authoritative domestic and international textbooks in the field, it has been found that such materials can generally be categorized into two types: one with a comprehensive focus and the other with a career-oriented emphasis. Given the orientation of this course as an academic English course, only the former category is referenced. The Table 8 below shows 5 representative books published in China and abroad for the purpose of comparing the themes and other linguistic units or academic tasks contained in them.

**Table 8:** Five representative books published in China and abroad.

1 (A monograph published aborad)	2 (A monograph published aborad)	3 (A monograph published aborad)
**The Story of Architecture: From Antiquity to the Present**(**Jan Gympel)**	**Architecture: From Prehistory to Postmodernism** (**Trachtenberg & Hyman)**	**A World History of Architecture (Michael Fazio, Marian Moffett & Lawrence Wodehouse)**
**ANTIQUITY AND EARLY CHRISTIANITY (2900 B.C. – 540 A.D.)**	**Part I The Ancient World**	Chapter 1 The Beginnings Of Architecture
Architecture in ancient Egypt	1. Architecture Before Greece	Chapter 2 The Greek World
Classical Greece & Hellenism	2. Greece	Chapter 3 The Architecture Of Ancient India And Southeast Asia
The architecture of the Roman Empire	3. Rome	Chapter 4 The Traditional Architecture Of China And Japan
Early Christian & Byzantine architecture	**Part II The Middle Ages**	Chapter 5 The Roman World
**ISLAM**	4. Early Christian and Byzantine Architecture	Chapter 6 Early Christian And Byzantine Architecture
From Muhammed to the fall of Granada Google	5. Pre-Romanesque and Romanesque Architecture	Chapter 7 Islamic Architecture
The great Ottoman era	6. Islam and the West	Chapter 8 Early Medieval And Romanesque Architecture
**THE ROMANESQUE PERIOD**	7. Gothic Architecture	Chapter 9 Gothic Architecture
Carolingian & Ottonian architecture	**Part III The Renaissance & the Baroque**	Chapter 10 Indigenous Architecture In The Americas And Africa
High Romanesque under Salians and Hohenstaufens	8. The Renaissance	Chapter 11 Renaissance Architecture
Alternatives to Imperial Architecture	9. The Baroque	Chapter12 Baroque Architecture
**GOTHIC**	**Part IV The Modern World**	Chapter 13 Neo-Classicism, Romanticism, And The Rococo
Classical cathedral Gothic in France	10. The Eighteenth Century	Chapter 14 Eclecticism, Industrialization, And Newness
Gothic in England	11. The Nineteenth Century	Chapter 15The Twentieth Century And Modernism
Gothic in Germany	12. Modern Architecture	Chapter 16 Modernisms In The Mid- And Late Twentieth Century And Beyond
Gothic in Italy	13. Second Modernism (through Post-Modernism)	
**RENAISSANCE**	14. Modernisms: Renewal and Hyper-Diversity in Recent Decades	
Florence & the early Renaissance		
High & Late Renaissance (Mannerism)		
Renaissance north of the Alps	4 (A textbook published in China)	5 (A textbook published in China)
**BAROQUE & ROCOCO**	**English for Architecture (Heng Xiaohan)**	**Chinese Architecture: Palaces, Gardens, Temples and Dwelling** (**Yanxin Cai)**
Emergence of Baroque in Italy	**PART 1 — History of Western Architecture**	
Palaces & Gardens in France	Unit 1−1 A Lesson on the Word Architect	Ancient Cities
Baroque in Germany & Rococo	Unit 1–2 Ancient Greek and Roman Architecture	The Supreme Imperial Power
England and trend toward Classicism	Unit 1–3 Medieval Architecture	The Palaces of Gods
**CLASSICISM**	Unit 1–4 Gothic Architecture	Appreciation of Chinese Gardens
Enlightenment & revolutionary architecture	Unit 1–5 Renaissance Architecture	Vernacular Dwellings
Classicism as state architecture	Unit 1–6 19th-Century Western Architecture	When East Met West
**HISTORICISM & INDUSTRIAL ARCHITECTURE**	Unit 1–7 New Art and Architecture	
Historicism	**PART 2 — Chinese Architecture and Culture**	
Industrial architecture	Unit 2−1 Distinctive Features of Chinese Architecture	
The Chicago School	Unit 2−2 Urban Planning of Ancient Chinese Cities	
**THE FIRST HALF OF THE 20TH CENTURY**	Unit 2–3 Imperial Architecture of Ancient China	
The search for a new form	Unit 2–4 Vernacular Architecture in China	
International style/Rationalism	Unit 2–5 Chinese Gardens	
**THE SECOND HALF OF THE 20TH CENTURY**	Unit 2–6 Preservation of Architectural Heritage in China	
The triumph of modern architecture	Unit 2–7 Global Influence of Chinese Architecture	
Sculptural architecture	**PART 3 — Practice of Architectural Design**	
High-tech architecture	Unit 3−1 Advent of Modern Architecture	
Post-modernism	Unit 3−2 Visions of Computer-Aided Architectural Design	
Deconstructivism & trends	Unit 3−3 Architecture Competitions	
	Unit 3–4 Architecture Design Process	
	Unit 3–5 Architecture Drawings	
	Unit 3–6 Architectural Presentation	
	Unit 3–7 New Trends in Architecture	

First, it is evident that these books generally adopt a chronological or stylistic structure, incorporating case studies for illustration. The table of contents share major architectural periods and styles from both Eastern and Western history, including: ancient Chinese cities, imperial architecture, religious buildings, vernacular architecture, and gardens; as well as Ancient Greek, Ancient Roman, Early Christian and Byzantine Architecture, Pre-Romanesque and Romanesque Architecture, Islamic architecture, Gothic, Renaissance, Baroque, Industrial Revolution, 20th-century, and contemporary architecture, totaling 16 units. Therefore, the input units will utilize this structure and the materials for these sections will be drawn from renowned international monographs and key textbooks.

Second, an analysis of the unit structure in domestic textbooks reveals a consistent composition comprising the following components: 1) Texts: Each unit typically includes at least one intensive reading article and one extensive reading article. The selected texts are from international monographs as well as academic papers. For instance, the intensive reading text “A City Is Not a Tree” in each unit of the textbook *English for Architecture* authored by Jiang Shan [29] is from the important paper by architect Christopher Alexander [30]. 2) Exercises: Practice sections such as “Tips for Translation,” “Oral Presentation,” “Writing,” and “Listening” are included. This study will also consider employing those arrangements but with adjustments in both content and exercises.

#### 9.2.2 Five-move framework of texts in monographs.

This study selected as an example a text from the first part of Chapter 4 of *The Story of Architecture:* *From Antiquity to the Present* by Jan Gympel [31], which focuses on “Classical Cathedral Gothic In France 1130–1300.” Through textual analysis, it was determined that it should be put into the category of descriptive writing and academic works of this type on architecture typically follow a five-move structure:

Move 1: Establishing the Significance of the Field. The text begins by exploring the origins and definition of the Gothic style, directly establishing its core position in architectural history and its academic discourse value by introducing classic academic debates, the significance of the topic is highlighted.

*“It is extremely difficult to put an exact date on the transition from Romanesque to the Gothic style. Art historians fondly engage in disputes...”*

Then, the author directly emphasizes the cathedral as the epitome of Gothic architecture, establishing its representativeness with the line:

*“Of all building types, the cathedral is seen to epitomize Gothic architecture to the present day.”*

Move 2: Presenting core concepts. The text systematically introduces key technical features and components of Gothic architecture, such as “pointed arch rib vaults”, “flying buttresses”, “clerestory”, “triforium”, and “tracery”, to provide clear explanations.

Move 3: Explaining principles and effects. This is the core part of the text, which elaborates on how the technical innovations of Gothic architecture achieved their artistic effects. For example:

*“It was possible to build the ribs first and then fill in the surface between the ribs (the cells).”*

*“Pointed arches exert a weaker lateral thrust than the round-headed arches...”*

This explains the structural advantages of pointed arches. For each feature, the text tells the impact it produces. For instance, the following sentence shows the effect of animal decorations on the spiky pinnacle of Gothic Cathedrals.

*“Animal figures also featured extensively in the decorations, with the result that the entire church became an image of a paradise-like habitat for all types of living species.”*

Move 4: Exemplification. The text consistently uses specific architectural case studies to support and demonstrate its viewpoints, which is one of its most prominent features. This article listed 4 key pieces of architecture including: St. Denis, Sainte-Chapelle, Chartres Cathedral and Notre-Dame, Paris.

Move 5: Summarizing. The text completes the text by revealing the aesthetic and philosophical connotations of the Gothic style, naturally linking technology, art, and social thought.*“The new technical possibilities are linked in ‘God’s skyscrapers’ (Le Corbusier), with the vision of creating a* colorful imaginative representation of Heavenly Jerusalem.”

To test the generalizability of this five-move structure, two additional texts from architectural history books were analyzed: one on ancient Egyptian architecture and one on Baroque architecture.

The Chinese chapter, chosen from a monograph published in China [32], on the planning of ancient capital cities, exhibited the full five-move structure. It began by establishing the significance of capital city location designation throughout Chinese history (Move 1), introduced the Zhou dynasty’s city planning principles and the fiefdom allocation strategy (Move 2), explained the philosophical basis “taking the middle path” for symmetrical layout (Move 3), provided examples of inner and outer city wall systems (Move 4), and concluded with the purpose of this multi-layered design: to protect rulers and civilians alike (Move 5).

The chapter on Baroque architecture, drawn from a monograph [31], followed a similar rhetorical pattern. It opened by defining the term “Baroque” and establishing its historical context, noting that it was “initially used to define something which was seen as oddly shaped and tasteless” (Move 1). It then introduced core architectural features such as concave-convex facades, broken pediments, and oval windows (Move 2), explained the philosophical shift from the Renaissance circle to the Baroque ellipse as a symbol of a “melancholy attitude to the transitory nature of this life” (Move 3), and provided detailed case studies including Bernini’s St. Peter’s Square and the Palace of Versailles (Move 4). The text also included concluding reflections throughout, such as the observation that “feudal society mustered all its splendour for the last time” (Move 5).

These analyses suggest that while concluding parts may vary in its content, architectural history writings generally follow the same pattern in terms of the first four moves. This structure therefore provides a reliable framework for designing reading tasks, where students are guided to identify these moves in each unit’s intensive reading text. Following this principle, the analytical results of other genres will be systematically incorporated into their respective units.

### 9.3 Analysis of academic paper abstracts

A corpus of 15 abstracts from leading architecture journals was analyzed to identify the rhetorical structure of this genre. The analysis revealed a consistent four-move pattern, though occasionally with variations in sequencing.

A representative example is the following abstract from a study examining neighborhood sustainability in Cairo [33]:

Move 1: Establishing the research context.

*“Successful urban regeneration accentuates the major purpose of the built environment in achieving urban renewal, in particular preserving local identity and sense of place, promoting mixed-use communal living, and optimizing resources on a long-term plan. The city of Cairo, Egypt, has witnessed a significant urban growth, with new cities located in the urban fringe. However, it is questionable whether the neighborhoods located in the new expansions are regenerative and sustainable as claimed.”*

Move 2: Identifying the research purpose.

*“This study tackles the issue of evaluating new Egyptian neighborhoods’ sustainability.”*

Move 3: Describing the methodology or approach.

*“An extensive analysis was carried out for three Neighborhood Sustainability Assessment Tools (NSAT), LEED®ND, BREEAM Communities and CASBEE-UD, to address the most applicable one to the Egyptian context.”*

Move 4: Stating findings and conclusions.

*“The research showed that 1) the social dimension was poorly addressed together with 2) the difficulty of applying the tool in the Egyptian context, as it has different cultural and demographic characteristics. The research highlights the importance of addressing neighborhood livability in NSATs as well as the need for their adaptation to local context. This represents a solid foundation for future research and creating NSATs adapted to the Egyptian cultural context.”*

The abstract follows a clear logical progression. This structure was observed consistently across the corpus, present in almost all abstracts.

This four-move structure directly informed the design of Unit 18 (Academic Abstracts Writing). In this unit, students are first guided to identify each move in sample abstracts through structured analysis tasks. They then practice writing each move using sentence patterns derived from authentic examples. Finally, students produce their own abstracts for their studio projects or research papers, with peer feedback focusing on the presence and effectiveness of each move.

Due to space limitations of the journal article format, the remaining five genres identified in the course framework (case studies, lecture videos, design statements, and presentation scripts) are not dealt with here and the literacy part is simplified to the same term “Analyzing organization and language features”.

### 9.4 The architectural art English syllabus: An integrated framework

#### 9.4.1 Course structure.

Referencing the questionnaire findings, the distribution of content and activities is prioritized as follows: professional knowledge > reading > speaking > listening > writing. Based on this prioritization and insights from domestic and international teaching materials, the course is divided into two parts.

Part I: Architectural Knowledge Around the World (16 units) covers architectural history from both Eastern and Western traditions. The domestic section includes 5 units on Chinese ancient cities, imperial architecture, religious architecture, vernacular architecture, and gardens. The international section includes 11 units spanning from Ancient Greece to contemporary architecture.

Part II: Architectural Design Practice (3 units) focuses on output genres: design statements (Unit 17), research abstracts (Unit 18), and design presentation scripts (Unit 19).

#### 9.4.2 Unit design.

Each unit in Part I includes one intensive reading text (drawn from architectural monographs or textbooks) to develop professional knowledge and reading skills, plus one supplementary reading selected from academic papers, case studies, or lecture transcripts—approximately 6 papers, 5 case studies, and 5 transcripts distributed across the first 16 units. The units in Part II include materials from online sources, comprising two design statements from architecture websites, two research abstracts from academic journal websites, and two transcripts of architecture student presentation videos from YouTube.

Task design responds to students’ high demand for speaking practice, with oral activities integrated into most units. Except for the writing-focused units (17–19), each unit includes scaffolded speaking tasks progressing from simple to complex, alongside activities such as vocabulary mapping, mind maps, and note-taking.

The final result is shown in Table 9.

**Table 9:** Syllabus for the course Architectural Art English.

	Units	Text 1	Text 2	Literacies	Tasks
**Part I —Architectural Knowledge around the world**	Unit 1 Chinese Ancient Cities	Extracts from books on architecture	Extracts from literature	The 5 Moves of the monograph articles	(1) Knowledge: Draw a vocabulary map (palace → axis, hierarchy, courtyard, Forbidden City)
	Unit 2 Imperial Palaces	Extracts from books on architecture	Extracts from literature		(1) Divide the paragraph structure (2) Speaking: Orally summarize the three major characteristics of a palace as a group and provide examples.
	Unit 3 Religious Architecture in China	Extracts from books on architecture	Extracts from literature		(1) Create a mind map (2) Group discussion: “How does religious architecture reflect belief and culture?” followed by a 2–3 minute presentation.
	Unit 4 Vernacular Architecture in China	Extracts from books on architecture	Extracts from literature		(1) Find a video or images of a vernacular architecture case, give a brief introduction, and explain its cultural and functional significance.
	Unit 5 Chinese Gardens and Landscape Architecture	Extracts from books on architecture	Extracts from literature	Understanding research paper structure and language	(1) Identify the seven main sections of a research paper and take notes.
	Unit 6 Ancient Greek Architecture	Extracts from books on architecture	Extracts from literature		(1) List commonly used academic verbs and connectors in research papers, and practice translating example sentences.
	Unit 7 Ancient Roman Architecture	Extracts from books on architecture	Case studies from books	Analyzing case study organization and language features	(1) Reading task: Mark the moves in a case analysis text, extract the background, features, and significance of the case. (2) Orally describe an ancient Roman building.
	Unit 8 Early Christian and Byzantine Architecture	Extracts from books on architecture	Case studies from books		(1) List commonly used specialized terminology, explanatory verbs, and comparative language in case analysis texts, and practice translating example sentences.
	Unit 9 Pre-Romanesque and Romanesque Architecture	Extracts from books on architecture	Case studies from books		(1) List commonly used causal connectors, descriptive adjectives, and temporal and contextual expressions in case analysis texts, and practice translating example sentences.
	Unit 10 Islamic Architecture	Extracts from books on architecture	Case studies from books		(1) Create architectural feature description cards to analyze an Islamic building (2) Speaking practice: Organize language to explain the content of the cards.
	Unit 11 Gothic Architecture	Extracts from books on architecture	Case studies from books		(1) Speaking practice: Compare the form and function of Gothic and ancient Roman architecture, and analyze their cultural significance.
	Unit 12 Renaissance Architecture	Extracts from books on architecture	lecture transcript	Analyzing lecture organization and language features	(1) Identify the macro-structure of a lecture (2) Record the core content of the lecture.
	Unit 13 Baroque Architecture	Extracts from books on architecture	lecture transcript		(1) Identify example sentence patterns of moves (2) Write example sentence patterns.
	Unit 14 Architecture in the Industrial Revolution	Extracts from books on architecture	lecture transcript		(1) Learn expression patterns from lectures and practice imitating them orally.
	Unit 15 20th Century Architecture	Extracts from books on architecture	lecture transcript		(1) Writing task: Write a lecture summary, extract key points and provide comments.
	Unit 16 Modernisms In The Mid- And Late Twentieth Century And Beyond	Extracts from books on architecture	lecture transcript		(1) Watch a lecture video and take notes.
**Part II— Architectural Design Practice**	Unit 17 Design Statement Writing	Articles from the Architecture Competition and Portfolio website	Articles from the Architecture Competition and Portfolio website	Understanding design statement structure and purpose	(1) Provide students with a project page and have them extract the “Project Description / Design Statement” section to annotate the moves.(2) Students extract keywords, design concept sentences, connectors, etc., from the design statement and practice vocabulary and sentence patterns.(3) Ask students to imitate this structure and write a short design statement (150–200 words) for their own design or a class exercise.
	Unit 18 Academic Abstracts Writing	Academic journal articles, international academic conference websites	Academic journal articles, international academic conference websites	Four-move structure of academic abstracts	(1) In pairs: Student A orally introduces their research or interest project, Student B asks questions and takes notes. Student B then writes a short piece of abstract draft based on the information heard. Afterwards, switch roles. (2) Abstracts editing: The teacher provides a poorly written student abstract (e.g., too long, no results, lack of coherence) and has students work in groups to revise it.
	Unit 19 Architectural Design Presentation	Script of videos from Youtube	Script of videos from Youtube	Organizing and delivering design presentations	(1) Students introduce a design idea in 1 minute (could be a small class case or their own work). Must include purpose, one key feature and expected impact. (2) Case-based task: Provide a famous building (e.g., Fallingwater or Beijing National Stadium) and have students prepare a 2-minute presentation answering: What is the design concept? What problem does it solve? What feature is most striking?

## 10 Conclusion and future research direction

### 10.1 Conclusion

Based on the comprehensive analysis presented, this study outlines a research framework for designing the syllabus of an English course for art colleges and elaborates on the process in detail. The research commenced with a questionnaire survey to gather opinions from three groups within the institution: undergraduates, postgraduate students, and faculty.

The most significant finding of the study concerns the negative impact of a lack of professional knowledge on learning outcomes. This suggests that an EAP course cannot be simply reduced to a generic English language skills class. The teaching of professional knowledge may need to form a core component of EAP courses, which could pose a substantial challenge for English language instructors. The slow development of EAP courses in the domestic context might be largely attributed to this very obstacle. Furthermore, the survey revealed a strong communicative intent among the student group, including aspirations for studying abroad and interacting with international peers in their field. This points to the importance of prioritizing speaking, a productive language skill, which could be adequately reflected in teaching activities.

Additionally, the survey identified a general underestimation of the importance of listening and writing skills, a common phenomenon in English language education. Since listening and speaking are inherently interconnected skills, improving oral proficiency necessarily involves enhancing listening comprehension. As for writing, the questionnaire data indicated it was perceived as the least important skill; consequently, only limited space was allocated to it in the course design. However, its relative importance warrants would benefit from further investigation and confirmation through additional research.

Utilizing the genre analysis model proposed by Swales, this study examined relevant domestic and international architectural textbooks, monographs, research papers, and other materials to design the overall content framework, unit themes, and module structures. Firstly, by consulting architectural reference materials as well as the results of the questionnaire, two broad genre categories were established: input and output genres. These were further subdivided into four input genres: textbooks and monographs, research papers, case studies, and lecture scripts and three output genres: design statements, presentation scripts, and research abstracts. Based on the survey results and genre analysis, corresponding teaching content, along with post-class exercises and activities, were developed for each unit.

This study’s primary theoretical contribution lies in the systematic integration of Dudley-Evans & St John’s [4] needs analysis framework with Swales’ [13] genre theory in syllabus design. While previous research has applied these frameworks separately—using needs analysis to identify what students need to learn, or genre analysis to describe how disciplinary texts are structured—this study demonstrates how they can work in tandem. Needs analysis provided empirical data on students’ target needs, lacks, and subjective preferences, guiding the selection of genres and the weighting of skills across the syllabus. Genre analysis revealed the move structures and rhetorical conventions of key architectural texts, shaping the sequencing of unit themes and the design of literacy tasks. Their integration helps ensure that the resulting syllabus is both responsive to learner needs and authentically grounded in the communicative practices of the architectural discipline—a theoretical advancement over approaches that rely on only one of these perspectives.

### 10.2 Limitations and future research direction

Several limitations of this study should be acknowledged. First, due to space limitations and the methodological focus on representative sampling for syllabus design purposes, this study provides detailed move-step analyses for only two genres—textbooks/monographs and academic paper abstracts—as illustrative examples of the genre analysis approach. The remaining five genres identified in the course framework (case studies, lecture videos, design statements, research abstracts, and presentation scripts) were not included in the detailed analysis, as the purpose of this study is not to exhaustively analyze all genres, but to identify representative rhetorical patterns that can inform syllabus construction. Future research will extend the genre analysis to these additional genres, providing a more comprehensive understanding of the rhetorical conventions that characterize architectural discourse.

Second, this study was conducted at a single institution in China. While the sample size was large, the findings and the resulting syllabus may not be directly generalizable to other educational contexts. The results may not apply to comprehensive universities with different curricular priorities. The findings may not transfer to art institutions in other countries with different educational systems. Future research should replicate this study across multiple institutions to determine the extent to which these findings can be generalized.

Third, the study relied on self-report questionnaire data, which is subject to potential biases and inaccurate self-assessment. Students may have overestimated or underestimated their actual abilities or needs. Future studies could complement self-report data with objective measures such as language proficiency tests, classroom observations, or analysis of student work.

Fourth and most importantly, the present study is primarily descriptive in nature, focusing on the design and development of the syllabus based on needs and genre analysis. The proposed syllabus has not yet been implemented or validated in actual classroom settings. Therefore, future research should pilot the syllabus in real classroom contexts, collect data on student performance and engagement, and empirically evaluate its effectiveness on learning outcomes.

Despite these limitations, the study offers a replicable methodological framework for integrating needs analysis and genre analysis in EAP syllabus design, particularly in under-researched disciplinary contexts such as art and design education. Future research should also apply this framework to other specialized fields such as English for Design or English for Music to test its robustness and adaptability.

## Supporting information

S1 File Questionnaire for EAP NA survey. (DOCX)

S1 Table Original collected data. (XLSX)
