# Supplementary material for: Designing the syllabus for the EAP course “Architectural Art English”: A needs and genre analysis-based approach
Source: PLoS One. 2026 Jun 16;21(6):e0351750. doi: 10.1371/journal.pone.0351750 (PMC13271476; doi:10.1371/journal.pone.0351750)
Supplement: S1 File — (DOCX) [file pone.0351750.s001.docx]

**Questionnaire for** **EAP NA survey**

Thank you for participating in this survey questionnaire. This questionnaire aims to collect demands regarding the teaching content of the “Architectural Art English” course. Your feedback will provide extremely valuable references for our future course design, improvement of teaching methods and optimization of related teaching resources. This survey is completely anonymous. You can choose to stop filling out the questionnaire at any time without any consequences. If you choose to complete and submit the questionnaire, we will consider it as your consent to the use of your data in future research results. We attach great importance to your opinions and suggestions. Thank you for your time and support!

Please answer each statement based on your true opinions and experiences. Your responses will be kept confidential.

For statements in Sections 2 and 4, you will be asked to indicate the level of importance using a five-point scale.

5 - Very Important: This aspect is extremely important to you.

4 - Important: This aspect is generally important to you, though not essential.

3 - Neutral / Not Sure: You are unsure or feel neutral about the importance of this aspect.

2 - Not Important: This aspect is of little importance to you.

1 - Not Important at All: This aspect is completely unimportant to you.

For statements in Sections 3 and 5, you will be asked to indicate your level of agreement using a five-point scale.

5 - Strongly Agree: You completely agree with this statement.

4 - Agree: You generally agree with this statement.

3 - Neutral / Not Sure: You are unsure or have no strong feelings about this statement.

2 - Disagree: You generally disagree with this statement.

1 - Strongly Disagree: You completely disagree with this statement.

1. Please choose your identity.

Undergraduate freshmen

Graduate students

Teachers teaching architecture, landscape architecture, art and related fields

1. During the learning process in architecture, landscape architecture, and art-related majors, students often use English in various academic activities. Please evaluate the importance or frequency of the following English usage scenarios.
   1. Reading English academic journals
   2. Reading English research papers
   3. Attending professional courses taught in English by Chinese instructors
   4. Attending courses taught by foreign instructors
   5. Listening to lectures by foreign experts in English
   6. Orally analyzing famous cases in English
   7. Orally presenting one's own design work in English
   8. Communicating with international students, foreign instructors, or foreign experts in English
   9. Writing design descriptions for works in English
   10. Writing paper abstracts in English
   11. Writing short essays or reports for course assignments in English
   12. Students having needs for studying abroad or visiting scholar programs for further education
2. In the learning process of architecture, landscape architecture, and art-related majors, what difficulties do students typically encounter? Please select according to the severity of the problem. (Teachers select based on your own observations; students select based on your own circumstances.)
   1. Students find it difficult to read English textbooks, journals, and papers.
   2. Students cannot keep up with the speech speed or understand the terminology when listening to lectures, courses, or videos by foreign experts.
   3. Students have difficulty expressing themselves in English during class discussions, group presentations, design explanations, or when communicating with teachers and peers.
   4. Students have difficulty writing English design descriptions, paper abstracts, English reports for class assignments, etc.
   5. A major learning difficulty for students is the lack of professional English vocabulary and expressions related to architecture, landscape architecture, etc.
   6. A major learning difficulty for students is the lack of professional knowledge in architecture, landscape architecture, etc., leading to insufficient content in their English expressions.
3. What proportion do you think the following teaching content should occupy in the "Architectural Art English" course? Please rate them according to their importance.

4-1 Learning the basic knowledge of architecture and landscape architecture.

4-2 Mastering the core vocabulary and common terminology of architecture and landscape architecture.

4-3 Learning effective reading methods for English textbooks and professional journals.

4-4 Understanding and analyzing the structure and content of English papers in the field of architecture and landscape architecture.

4-5 Training listening comprehension of professional terminology by listening to lectures and watching videos.

4-6 Analyzing international architectural or landscape design cases in English, and elaborating on design concepts.

4-7 Training for design project presentations and defenses in English, enhancing the logic and professionalism of expression.

4-8 Organizing group discussions to practice design criticism and exchange of views in English.

4-9 Writing clear and structurally standardized English design descriptions.

- 1. Writing paper abstracts.
  2. Writing English report assignments

1. Which of the following teaching methods do you think should be adopted in the course? Please select according to their effectiveness. (Teachers select based on your own observations; students select based on your own preferences.)
   1. Teacher-led offline lectures.
   2. Blended online and offline teaching.
   3. Case analysis: analyzing classic or international architectural project cases to understand their design concepts and expression methods, thereby enhancing the practical application ability of professional English in architecture.

5-4 Design presentation simulation: simulating design presentation scenarios in class or competitions, training students' ability to introduce design concepts, methods, and highlights in English.

5-5 Group collaborative presentations: organizing students to conduct English resource research, project presentations, and outcome reporting in groups, honing teamwork and English expression skills.
